# Virtual emotions and Criminal Law

**DOI:** 10.3389/fpsyg.2023.1260425

**Published:** 2023-10-31

**Authors:** María Isabel González-Tapia

**Affiliations:** Department of Civil, Criminal Law, Procedural Law, Faculty of Law and Business, University of Córdoba, Córdoba, Spain

**Keywords:** virtual reality, Criminal Law, emotions, metaverse-crimes, neurolaw, neurorights

## Abstract

This article examines the role that Criminal Law should play in regulating the non-therapeutic use of immersive Virtual Reality (VR), specifically its massive use by consumers. The starting point has been to consider VR as an intermediate risk scenario, for the purposes of Criminal Law, between the criminality entirely generated in the physical world and that developed in the 2D digital environments [cybercrimes and criminality linked to social networks and persuasive Artificial Intelligence (AI)]. Firstly, specialize literature has been analyzed to establish the nature of virtual reality. From a technical standpoint, virtual reality is a neurotechnology infused with high-risk artificial intelligence; an inseparable synthesis of non-invasive neurotechnology and a set of AI systems, considered high-risk for the fundamental rights of citizens. From the perspective of its functioning, VR is a “transformative” neurotechnology capable of altering what people perceive as reality. This is possible, because its realism lies in the emotional immersion of the user in the virtual experience, similarly to how our brain functions. Therefore, the key idea in the immersive functioning of virtual reality is its capacity to evoke and modify human emotions, which results its greater harmful potential compared to the 2D environment. From there, three central and specific areas of (legally unaddressed) risk arise: (1) the special comprehensive nature of the data collected and stored during its use; (2) its ability to mentally reproduce the “physical” experience of the avatar in the user; and (3) its significant capacity to manipulate individuals. Secondly, the paper examines both the reported cases and the foreseeable criminality in virtual worlds or “proto-metaverse,” focusing on the three risk areas, and exemplifying them with attacks on mental privacy, sexual freedom, and consumer manipulation. Finally, it is proposed that Criminal Law should also intervene (as soon as possible) to define the “red lines” of massive virtual reality use by citizens. With a democratic and human-centered approach, a basic legal framework is outlined for the criminalization of specific harms and risks associated with virtual reality, adapting the existing legal framework as necessary.

## 1. Introduction

### 1.1. Criminological context: from cybercrimes to criminality through persuasive artificial neuro-intelligence

In the last quarter of the 20th century, Western Criminal Law began to undergo an expansive transformation. It was a process in response to the new social, political, and economic context of the so-called “risk society” (Beck, 1986; Beck, 2006), which brought with it new risks linked to industrial and technological progress and globalization. Unlike the traditional scope of liberal Criminal Law, these new risks were collective, with a global or systemic harmful potential. They were uncertain, medium to long-term, and usually involved the accumulation of multiple behaviors by non-coordinated individuals (*Cf.* Mythen, 2014). As Silva Sánchez (2001) describes, the Criminal Law expanded its protection to a significant number of collective legal interests (e.g., the environment) and anticipated its intervention before harm occurred, through offenses based on risk and the omission of control duties. While Criminal Law was still in an adjustment process, this initial risk society has been rapidly transformed, due to the impact of factors, such as the digital revolution, astonishing advances in neuroscience, and the current general disruption driven by artificial intelligence. And, again, this revolution occurring in society appears to have profound implications for Criminal Law, as an instrument aimed at social control and the protection of basic values and principles of our social coexistence.

For the sole purpose of situating the evolution of criminality in the digital context, we will consider that Web 2.0 corresponds to a scenario in which, in addition to accessing content disseminated on the internet (Web 1.0), it evolves into a dynamic, collaborative, and social platform, where users can interact and create content. This digital environment includes blogs, wikis, social networks, file hosting services, online payment services, and various online services. The criminality 2.0 was, initially, the so-called cybercrime, defined by the Budapest Convention on Cybercrime (Council of Europe, 2001) as illicit behaviors committed through (or facilitated by) the use of computer systems and information and communication technologies (ICTs). The adaptation of Criminal Law required updating its content to this new context, with new legal assets (e.g., data and computer systems) and new forms of electronic attacks on pre-existing legal rights (e.g., privacy or property). Additionally, cybercrime also demanded the establishment of an international strategy for an effective law enforcement, given the borderless space of the internet. In any case, with or without computer media, offenders and victims were just physical or legal persons, and crimes had their expression in the physical world, with real damage to goods and services (Miró-Llinares, 2012). Therefore, despite significant difficulties in adjustment, the context remained recognizable for Criminal Law policy.

In a second phase, in the transition to Web 3.0, another factor has been added. In addition to the computing and the internet, the interactive factor has been incorporated, leading to new forms of social-media criminality, committed through (or facilitated by) the use of social networks and to an intensification of the potential risk in pre-existing attack methods. For example, social networks, besides challenging users’ privacy, have multiplied the harmful potential of attacks on personal assets, due to their capacity for massive and immediate dissemination of content (“virality”) and the possibility of public interaction among users. This has led to a proliferation of expression-related offenses through audiovisual media, such as cyberbullying, hate speech, public defamation and/or woke-based cancelations, which not only directly damage an individual’s honor or self-esteem, but also indirectly stifle their freedom of expression. For the Criminal Law, in addition, another specific problem arises, regarding the by accumulating-responsibility of multiple non-coordinated users, who re-share or simply comment on such illicit content, thus amplifying the harm caused by the offense (*Cf.* Agustina, 2021).

In the next step, the digital environment has evolved into the Web 3.0, which could be understood as the result of the incorporation to the digital environment of artificial intelligence and the so-called semantic web, both driven by the convergence of the neurotechnological revolution (Savage, 2019). In this environment, persuasive AI (Fogg, 2002) plays a leading role along with extended reality, including virtual, augmented or mixed reality, among other variants (Mann et al., 2018). If we simplify, while the goal of Web 1.0 was content dissemination and Web 2.0 added interaction, the goal now is to create a more personalized, precise, and immersive environment and experiences. Regarding AI techniques, the evolution has progressed from “machine learning” to “deep learning,” based on algorithms that emulate the functioning of complex neural networks. Thus, considering only a basic scheme for the purpose of analyzing its legal implications, deep learning operates based on: (1) extraordinary computational capacity, enabling the management, processing, and analysis of vast amounts of data (“big data”); (2) equally extraordinary predictive ability, by detecting multiple patterns in the data and formulating predictions, subsequently storing the generated intelligence; and (3) unparalleled operational capacity, allowing for “massive” execution but also “personalized” actions, such as offering personalized and real-time advertising to specific consumers or consumer groups, based on acquired knowledge about their preferences, values, circumstances, and needs (Hermann, 2022). To perform these tasks, artificial intelligence relies on advanced algorithms fed by a myriad of data, which need to be increased (in terms of quantity, diversity, and timeliness) the more complex the problems to be solved or the more variables influencing the definition of a strategy or any decision. In summary, the raw material of the entire economy underlying Web 3.0 are “data”; vast amounts of semi-processed data that must be supplied to the AI system for its functioning: macro-data or “big data.” It is not surprising, therefore, that the term coined to express the new capitalist model arising from the need for information as a priority and highly valuable economic resource is “surveillance capitalism” (Zuboff, 2019). On the other hand, as previously mentioned, the revolution of artificial intelligence converges with the equally remarkable revolution in neurobiology and its associated technologies. AI and neuroscience reciprocally feed each other in an inseparable synthesis, which allows, in the vivid expression of Rafael Yuste-El País 26/03/2023 (2023): “reading and writing” our brain (also Goering et al., 2021). In short, the circle is completed when it becomes technically possible to access the most intimate information of human beings and modify it. That is, changing beliefs, thoughts, and emotions of an individual and, from there, their behavior as well, using neurotechnologies.

I argue that, for the purposes of Criminal Law, this disruptive intersection of artificial intelligence with neurobiology is precisely the crucial element from which to extract a substantial difference from previous digital environments. It is the factor that has given rise to a new category of radical and systemic risks, derived, on the one hand, from the possibility of modifying human nature through implants and other non-invasive neurotechnologies (*neurointerventions*) and, secondly, from the possibility of manipulating the mental states of human beings and, therefore, their behavior. Moreover, it is also possible to engage in social engineering by manipulating the “mental states” of groups through algorithms designed to induce opinions. Obviously, it is not that the risk of manipulating human beings and social groups did not exist before; what has changed now is the effectiveness with which it can impact the population through neurotechnologies and persuasive artificial intelligence, as well as its covert or even subliminal nature.

### 1.2. Legal assumptions and delimitation

Firstly, it must be clarified that analyzing the criminal legal treatment of immersive virtual reality does not imply strictly analyzing the penal governance of the so-called Metaverse, which has been defined by Ball (2022, p. 29) as: “a massively scaled and interoperable network of real-time rendered three-dimensional (3D) virtual worlds that can be experienced synchronously and persistently by an effectively unlimited number of users with an individual sense of presence, and with continuity of data, such as identity, history, entitlements, objects, communications and payments.”

Currently, a comprehensive Metaverse does not exist. What exists today are proto-Metaverses, decentralized or proprietary, based on blockchain technology, such as Decentraland or Sandbox; virtual worlds owned by a platform, like Horizons World by Meta; and games like Minecraft or Fortnite, which offer virtual gameplay, spaces, and experiences. For our purposes, all of these are 3D virtual or immersive spaces with creative user participation and life-like social interaction in multiplayer mode. These characteristics are also inherent to the concept of the Metaverse, which has been described as shared/collaborative/social VR (Zhang and Hong, 2023). Therefore, we will refer generally to immersive virtual reality as the underlying and central technology in all these spaces and as encompassing the issues that may also arise in the theoretical Metaverse. Furthermore, as it is being ideally conceived, the current Criminal Law could not play any role as we know it. A global, decentralized, and independent Metaverse would be something akin to a new “fictional” world, with a parallel society having digital identity and governed by capitalist companies, which would ultimately own the Metaverse infrastructure. This simply implies a new social contract upon which to build the law. Only a Metaverse dependent on nation-states would have the authority to enforce governance by means of Criminal Law, given that the *ius puniendi* (the right/power to punish) is a prerogative of sovereignty, which so far remains tied to the concept of the State. A decentralized Metaverse with DAOs and secured by blockchain technology may be feasible and meaningful for the internal governance of virtual worlds, as proposed by Bermejo-Fernandez and Hui (2022). However, from the perspective of Criminal Law, Metaverse governance can only be considered externally and from the idea of legal dependency on the real world, in connection with countries’ rule of law. Therefore, a distinction must be made between the governance by the Metaverse in different spaces and the governance of the Metaverse, which is linked to country regulations and policies, as proposed by Marijn Janssen in Dwivedi et al. (2022).

It should also be noted that there are various levels of immersion to experience virtual reality, ranging from 2D screens and 360-degree videos to using goggles and headsets, and further enhancing the experience with different haptic devices. Similarly, there are different ways to experience reality, including augmented reality, mixed reality, and virtual reality. However, the most relevant issue for Criminal Law, and the focus of this study, is fully immersive virtual reality, as opposed to other intermediate experiences in which the user is still in contact with the real world, even if it involves first-person experiences interacting with virtual objects or a three-dimensional environment. As Rauschnabel et al. (2022) posits, Augmented Reality (AR) and VR have fundamental differences, as the former can be described as “local presence,” while the latter is conceptualized as “telepresence,” spanning a continuous range from atomistic to holistic VR. Holistic immersive experiences, highly realistic and available to consumers, are likely to generate the most significant risks (Slater et al., 2020) and, therefore, require prompt attention from Criminal Law. In any case, it should be acknowledged that the criminal treatment of a specific act, committed in a virtual space, will directly depend on its nature and the level of immersion of the experience. Not all levels of immersion should have the same legal treatment, as the degree of harm decreases significantly when the environment is not fully immersive, similar to what can be attributed to a 2D environment.

On the other hand, this study will not address the direct use of neurotechnologies for therapeutic purposes. As I have asserted in a previous work, the direct use of those neurotechnologies (in a narrow sense), invasive or not, must be limited to the therapeutic or medical field, covered by deontological rules and ensuring the risks through criminal law as well. Similarly, the use of immersive VR in a therapeutic context is also covered by deontological rules, that counterbalance its potential harm(*Cf.* for ethics concerns Madary and Metzinger, 2016). Any harm from its use in this context, if performed in accordance with the standard of practice (*lex artis*), is legally considered within the permissible risk (González-Tapia and Isabel, 2022). We will solely focus on immersive VR as neurotechnology, in a broad and functional sense, as a consumer technology.

## 2. Immersive VR as an emotion-induction neurotechnology

It could be said that VR is a high-tech human-computer interface in which, using different devices and connection to a computer or gaming platform, users are able to immerse themselves in three-dimensional virtual experiences. Screens and lenses are used for stereoscopic 3D vision, headphones and surround audio provide auditory immersion, motion sensors track head and body movement, and hand controllers enable touch, while haptic devices with vibrotactile signals (such as gloves, vests, or full-body suits) enhance the sense of touch. All these components work together to create the illusion for the user of being inside, being present, and interacting in a first-person, real-time virtual environment through an avatar. In contrast to augmented or mixed reality, the virtual environment is entirely digital and artificial (Huynh-The et al., 2022). Currently, the most used devices in society for virtual reality (VR) are head-mounted displays (HMDs) and headphones. As a result, VR systems have primarily focused on audio-visual immersion up to this point, which serves as a fundamental benchmark for immersion in this study (Slater and Sanchez-Vives, 2016).

The first question that arises is whether virtual reality can be considered a neurotechnology or not. This is a particularly relevant issue, considering that the international debate on neurorights is connected to the use of neurotechnologies on human beings (Ienca and Andorno, 2017; Yuste and De La Quadra-Salcedo, 2023, for all). If virtual reality is not considered a neurotechnology, the reinforced protection sought for neurotechnologies would not directly apply. From a technical standpoint, neurotechnology is defined by Ligthart et al. (2023) as a technology that either measures brain activity or structure (e.g., through fMRI or EEG) and/or interferes with brain activity (e.g., through brain-computer interfaces and brain stimulation). From this narrow sense, it would have to be concluded that VR is not a neurotechnology, because it does not use devices specifically designed to read or modify brain activity. However, if we focus on its functional dimension, as usual in Criminal Law, the question is no longer so straightforward. Virtual reality can read mental activity through a conglomerate of collected data, and, more importantly, it can directly influence that activity by, for example, generating emotions in the user and activating spatial memory. For this reason, it has been used in therapy for years (Gao et al., 2023), using the predictive functioning of the brain, artificially generating sensory input (audiovisual and haptic) and stimulating various neurobiological, psycho-emotional, and behavioral responses in the user, which can generate memories. From this perspective, as proposed by Vincent et al. (2020, p. 11) from a functional standpoint, VR could be considered a neurotechnology, because it has the direct ability to interfere with brain activity through the use of technology. However, it would still be a non-invasive neurotechnology with less efficacy than strict neurotechnologies that directly read and/or interfere with brain activity.

Regarding its operation, immersive VR cannot be separated from AI. From a technical standpoint, in addition to other haptic and external devices, fully immersive VR is made possible through an indissoluble synthesis of non-invasive neurotechnology (head-mounted displays) and a set of AI systems related to voice recognition, language processing, analysis of visual patterns, movement, facial expressions, emotions, and physical interaction for avatar control and environment interaction. The key technologies that contribute to the immersive experience and merging of the user with the virtual environment and avatar in virtual reality (VR), as well as in extended reality (XR) in general, include human interface technologies (such as mobile devices, smartwatches, smart glasses, wearable devices, head-mounted displays, gestures, voice recognition, and electrode bundles) and spatial computing technologies (such as 3D engines, geospatial mapping, and multitasking) (Huynh-The et al., 2022). Through the combination of these technologies, the user’s brain perceives being inside an alternative reality and experiences it firsthand, with the level of immersion and presence facilitated by the utilized devices.

In immersive VR, different non-invasive neurotechnologies are combined with other technologies and artificial intelligence to create, first and foremost, an immersive environment for the user. This means, creating an environment that allows the user to be fully immersed in it, with a high sense of realism. Users can experience virtual environments on two-dimensional screens, which provide a first-person experience but do not isolate them from the surrounding reality; or they can fully immerse themselves with head-mounted displays (HMDs) and headphones. Secondly, from a subjective perspective, immersive virtual reality aims to achieve a sense of active presence for the user in the virtual environment. It relies on the illusion of non-mediation, direct interaction, and the feeling of being located inside the virtual space (Slater, 2009). Clearly, this again depends on the level of immersion that each VR technology allows. Thirdly, another characteristic of virtual reality is the embodiment of the user or identification with their avatar. The user is made to feel that the avatar’s body is their own, experiencing the environment firsthand through it. Conversely, the avatar also influences the behavior and self-perception of the user during the experience, as described in the *Proteus effect* (Yee and Bailenson, 2007); or immediately after (Rosenberg et al., 2013). The embodiment of the user will be greater when audiovisual devices are combined with haptic devices, creating a more complete connection between the user and their avatar.

In terms of its effects, VR can be seen as a (de)constructive technology. VR is designed to give users *the feeling* of real lived-in experiences, using an effective emotion-induction technique, which provides a high degree of ecological validity combined with high experimental control.

In a 3D immersive experience, we perceive a first-person experience through perceptual illusion, spatial relocation, plausibility, and transformed agency. This becomes feasible, first, because virtual reality operates predictively, much like our brain does in its interaction with the real world. Neuroscience draws a comparison between our brain and a simulator that, through a lengthy evolutionary process, has acquired the ability to anticipate sensory stimuli before they are consciously perceived (predictive coding). Similarly, immersive virtual reality operates predictively, producing lifelike content that aligns with what our brain would generate in response to the real world (Riva et al., 2019). Secondly, because the features of immersive virtual reality intertwine cognitive perception of the virtual environment with induced emotions.

Literature is showing that the central strategy of VR is not only to provide high ecological validity to the digital environment (e.g., as seen in 360° videos). The basis of its realism lies in the *user’s emotional immersion* in that environment, transforming them from mere spectators into emotionally *engaged participants*. Slater et al. (2020) refer to this as *psychological realism* or illusion. The user has the psychological sensation that what happens in a VR world could be happening and is a real experience. Thus, immersion, presence, and avatar identification are enhanced when the experience is integrated with the capability to evoke emotions in the user, especially anxiety, relaxation, fear, and joy (Bernardo et al., 2021). Steinert and Friedrich (2020) differentiate two basic affective states: emotions and moods. They define emotions as intentional and motivational mental states because they involve a relationship between the person and something else (i.e., the object of the emotion) and are usually accompanied by bodily sensations. In contrast, moods are typically long-term, unintentional, and more diffuse. This emotional response is, once again, dependent on the user’s immersion, with significant differences in the induction of discrete emotions in VR-3D and Screen-2D modalities (Xie et al., 2023); along with greater brain activation in VR-3D and a greater immediacy (Tian et al., 2022b).

Consequently, immersive VR could be considered, in essence, an external affective neurotechnology, characterized by its ability to detect, influence, and stimulate affective states. It is also possible to accurately identify the specific stimulus that triggers an emotional response in real-time, determining whether the reaction is positive, negative, or neutral, along with its intensity and the behavioral expressions associated with it (Bar-Zeev, 2019). Additionally, by personalizing the approach, an interactive and reciprocal system can be established, capable of evoking specific emotions in an individual based on previously gathered information. In essence, as our understanding of human emotions expands and our ability to comprehend the complexities of an individual’s emotions improves, we gain the capacity to utilize this knowledge for intervention and manipulation of those emotions.

Moreover, VR (and AR) can activate highly plastic GPS neurons (Jayakumar et al., 2019) and make the subject perceive that they are truly present in the place projected by the virtual environment. Our motor, perceptual, and physiological systems are activated in the same way in immersive VR as they are in the real world (Bailenson, 2018, pp. 19–20). For this reason, it allows for the activation of spatial memory in our brain, creating memories of the experience that are difficult to distinguish from reality (Rubo et al., 2021). Additionally, Cadet and Chainay (2020) suggest that episodic memory is highly influenced by emotion, and in virtual experiences, this relationship is significantly mediated by the immersion provided by the device used.

For all these reasons, as Riva and Wiederhold (2022) posit, VR is not only a persuasive technology but also a transformative technology, capable of altering people’s perception of reality. And it is precisely this capacity that makes the metaverse significantly different from previous technologies. Hence, the numerous potential applications of virtual reality in the medical, educational, and recreational domains should not be surprising. Likewise, the noteworthy surge in its current utilization as it transitions from the laboratory to consumer technology should also be expected. Nevertheless, this potential widespread use is also precipitating significant uncertainties and ethical concerns.

VR is widely used for training and to promote mental health and personal well-being (*Cf.* Lindner, 2021), although there is still no consensus regarding its long-term effectiveness and consequences. For instance, concerning the treatment of anxiety and depression, the meta-analysis conducted by Fodor et al. (2018) found no significant differences compared to other active interventions. Similarly (Wong et al., 2023) found no significant benefits for social anxiety disorder. However, the meta-review conducted by Riva et al. (2019) concludes, positively, that the use of virtual reality in mental health is an established therapy, applied in the treatment of trauma, physical rehabilitation, phobias and anxiety disorders, eating disorders, and pain management, with long-term effects. Additionally, the meta-analysis by Wu et al. (2020) also concludes that the use of immersive VR can enhance both knowledge and skill development and maintain the learning effect over time. Similarly, in the context of consumers (Taufik et al., 2021). Regarding empathy, virtual reality is widely employed to foster empathy by enabling individuals to “inhabit” the experiences of others (“Virtual reality perspective-taking”) or, as commonly stated, to walk in someone else’s shoes, experiencing social exclusion, racial or age discrimination, gender or domestic violence, with positive outcomes in motivating prosocial behavior (Herrera et al., 2018). Nonetheless, its use and its long-term effects are a subject of debate (Franks, 2017; Rueda and Lara, 2020).

Moreover, other ethical concerns have also emerged. Firstly, in relation to lawful and authorized uses, because the intervention and modification of emotions, which naturally belong to and characterize the individual, should be self-managed as a principle (Steinert and Friedrich, 2020). Concerning the use of VR for moral enhancement (Rueda and Lara, 2020) have emphasized the necessity for rational oversight of its application, as it has the potential to result in morally incorrect behaviors and may not consistently guide us in moral dilemmas (see also Madary and Metzinger, 2016). Very importantly, the issue of potential dual use has also been noted, where these same technologies can be illicitly employed to manipulate our thoughts, perception of reality, preferences, and subsequently, our behavior. As pointed out by Bublitz and Merkel (2014), this raises significant concerns about misinformation and manipulation of users’ mental states, which have not yet received adequate attention in legal literature. And certainly, I concur with these authors that this is a highly relevant risk that should be examined from the perspective of Criminal Law. Furthermore, attention has also been drawn to the potential and lasting changes that prolonged use of immersive VR could generate in our brain, personality, and/or behavior, considering the functioning of this technology and neuroplasticity. Madary and Metzinger (2016, p. 4) highlight the significance of unconscious environmental influence on human behavior, particularly in the context of immersive VR, as it introduces an entirely novel environment that can effectively interact with the extensive array of human *epigenetic traits*. Similarly, concerning neurotechnologies in general, Wolpaw has cautioned that the issue with neurotechnologies lies in the artificial nature of the process, as it circumvents the established natural system of input and output. This artificiality may potentially lead to unpredictable changes in brain function (UNESCO, University of Milan-Bicocca, 2023, p. 15). Madary and Metzinger (2016, p. 13) assert with clarity: “We simply do not know the psychological impact of long-term immersion,” a risk that must be considered as VR becomes a consumer technology.

In conclusion, immersive VR is an external affective neurotechnology with transformative capacity, capable of altering individuals’ perception of reality, at least during the experience and immediately afterward. This is possible because its basis for realism lies in emotionally immersing the user in the virtual experience, operating in a manner like the functioning of our brain. Immersive virtual reality ‘transports you to the world within the screen.’ And it does so in a way that humans perceive as real, even though they know it is not, simply because the same cognitive and emotional mechanisms used in our interaction with the physical world are employed. From Section 2, it can also be inferred that the fundamental concept underlying immersive VR is its ability to evoke and alter human emotions (Markowitz and Bailenson, 2021). For this reason, emotion also needs to be central from a criminal policy perspective. Precisely, the management of emotions is what poses a greater risk to user rights, compared to the 2D environment. This increased risk arises because: (1) in immersive VR experiences the brain perceives VR as a genuine experience, potentially leading to the formation of memories that become entangled with reality; and (2) the comprehensive extraction and storage of information in the utilization of this technology result in the user’s transparency, in terms of their identity and inner self. As we will discuss bellow, this information encompasses biometric, emotional, and behavioral data.

To this point, however, the consequences on the brain, personality, and/or behavior, resulting from prolonged use of immersive VR, remain largely unknown. The long-term impact of immersive VR is not well understood, drawing comparisons solely from the long-term experiences in 2D environments. This circumstance adds another layer of complexity to the legal treatment of immersive virtual reality, because require a clear distinction between: immediate and direct harmful effects, that arise from the virtual experience; and potential, indirect and long-term harmful outcomes that are, in essence, still unknown. This long-term concern is related to certain aspects included in Section 3, referring us there for further consideration.

## 3. Specific risks of virtual reality and proposed legal treatment

In the realm of Criminal Law, the analysis highlights three primary and specific areas of risk in the foreseeable widespread of commercial immersive VR: (1) the comprehensive nature of the collected information during its utilization; (2) its capacity to mentally replicate the “physical” experience of the user’s avatar in the brain; and (3) its significant potential to manipulate individuals.

### 3.1. Risk 1: User privacy

One of the central problems arising from the convergence of artificial intelligence and neurotechnologies is the privacy of users’ mental data, due to its potential for human rights abuses (Ienca et al., 2022, for all). By its nature, user mental privacy is also a key issue in immersive VR. The psychological characteristics of immersive experiences pose an additional risk factor to users’ privacy for two reasons. First, unlike the ethical coverage that exists for the use of neurotechnologies in therapeutic contexts, VR is made available to the public for consumption, with the anticipation of mass consumption, opening the door to commercial and social control opportunities and a decrease in guarantees. Second, the combined nature of the data that can be collected through the various devices involved in the immersive experience. Through immersive virtual experiences, the real environment surroundings of the user can be captured, through geolocation, microphones, cameras, and sensors integrated into the devices (input privacy), but also: the user’s voice, eye biometrics, pupil activity, and other biological data, through haptic devices, while conversations and videos of the user’s activity in the virtual world can be recorded. Thus, the range of information that can be extracted, stored, and processed in the use of this technology is much broader than what can be obtained in a 2D environment and can impact the bystanders’ privacy as well (Roesner et al., 2021). It should be noted, however, that some of this information is directly provided by the user, influenced and uninhibited by the virtual context when interacting with other users. In fact, an online survey by Sykownik et al. (2022) shows that most users disclose sexuality-related information, lifestyle preferences, and personal goals, in contrast to identity-related information. These data allow for the inference of sensitive information regarding identity, race, sexual diversity, cultural traits, psychological profiles, potential diseases based on movements, and more (Qamar et al., 2023). They also provide a dynamic snapshot of the expression of mental states, primarily through the combined use of eye-tracking and pupil dilation technologies. Eye-tracking reveals where users look, what they focus on, and for how long, while pupil dilation shows their level of interest in specific stimuli. That’s why (Bar-Zeev, 2019) has referred to these techniques as “the advertising’s Holy Grail” and “an unconscious like bottom for everything...” “The closest thing we can imagine to your digital self.”

Very pertinently, Heller (2020) has proposed the concept of “Biometric psychography” as a new term for this type of dynamic body-centered information, not only linked to identity but mainly to interest and derived from eye-tracking and pupil response, facial scans, galvanic skin response, electroencephalography (EEG), electromyography (EMG), and electrocardiography (ECG). Spiegel (2018) also refers to it as a “kinematic fingerprint.” Essentially, the user becomes (or will become soon) “transparent” in their identity, various aspects of their biology, and their inner self. It is therefore easily understandable that various international organizations are warning about the relevance of this risk, especially in sectors such as neuromarketing (UNESCO, University of Milan-Bicocca, 2023), and the negative implications it can have for citizens’ social relationships and public freedoms (OHCHR, 2018).

Regarding its legal treatment, it is rightly pointed out by Heller (2020) that it is incorrect to treat immersive VR as a mere evolution of the risks associated with the 2D digital world. She argues that the psychological aspects inherent to immersive technologies make them not only quantitatively more dangerous but substantially different. The immersive nature of VR generates a response of psychological authenticity in the user, which also translates into a physiological response, similar to their bodily response in real situations. Considering this, the author proposes enhanced user protection by the industry through various measures: strengthening the user’s informed consent regarding their privacy and the immersive experience, establishing different levels of content moderation specification, and implementing a rating-based system for VR/AR entertainment or creating industry-wide codes of conduct.

In my opinion, this represents a crucial initial level of user protection and, quite possibly, the optimal approach for effectively safeguarding user privacy. Additionally, I acknowledge that the industry’s protective measures, encompassing risk assessments and corresponding mitigation measures such as compliance programs, are indispensable as they establish the foundation for potential civil or criminal liability in cases of crimes perpetrated on their platforms. However, I argue that it cannot be considered sufficient.

The second level of protection should be provided by regulations pertaining to the protection of personal data. However, both in the United States (Heller, 2020) and within the European Union (Spiegel, 2018), there are very significant loopholes and there is an urgent need to update this regulation. First, because the data collected during immersive VR does not fall under the category of particularly sensitive information, as defined in Article 9 of the General Data Protection Regulation (EU 2016/679) - GDPR, as they do not directly reveal ethnic or racial origin, political or philosophical opinions, trade union membership, or sexual orientation or life. Moreover, many times such data is disclosed directly by the user voluntarily. They are also not genetic data or biometric because, inexplicably, biometric data will only be those data “intended to uniquely identify” a person, and this category does not apply when the person is already identified or has given consent for such purposes. Second, because data protection laws have been built on isolated data and not on the idea of elaborated information, nor considering the predictive pattern-based operation of artificial intelligence. In virtual reality, the problem does not arise from isolated data but from the conglomerate of data, of diverse nature, provided by the immersive experience and processed with artificial intelligence systems. The central risk of virtual reality is that it directly provides a comprehensive and dynamic profile of the individual. Therefore, focusing solely on brain data to protect mental privacy is insufficient.

At a third level, the mental privacy of identified or identifiable users should also be guaranteed through criminal law. In my opinion, protection should be focused on three key aspects: (1) safeguarding structural and functional brain data as particularly sensitive personal information; (2) ensuring the requirement of valid informed consent, either initially or through the exercise of the right to erasure or the right to be forgotten (Articles 17 and 18 of the GDPR); (3) prohibiting the mass processing of mental data from identified or identifiable users, comparable to the prohibition of organ trafficking, to prevent their monetization.

Regarding neurotechnologies in a narrow sense, the *Morningside Group* suggests that, due to its biological nature of the signals carrying neural data, they should be protected by physical privacy and not be collected without informed consent (Goering et al., 2021). Similarly, but with a different justification, Wajnerman Paz (2021) also argues that neurological data should be protected as other organs, despite not being composed of organic material. The justification lies in the fact that neural data provide biometric information about a specific individual and, thus, reveals an identity trait. In the current technological landscape, where it is feasible to read brain activity and gain access a person’s thoughts and mental states, privacy legal protection needs to be updated. Neurological data should be recognized as particularly sensitive personal data and be subjected to an ultra-reinforced legal treatment, both as a personal legal asset within privacy offenses and as collective interest within the prohibition of human organ trafficking.

In the case of immersive VR, however, the issue becomes even more complex. Neural data, related to brain structure and functioning (endophenotype), are not directly collected, but rather mental information of the psychological phenotype. Mental states can be accessed and modified, influencing the resulting behavior. Therefore, the specific issue of mental privacy within immersive experiences lies in the psychological information that is collected through comprehensive real-time audiovisual, haptic, and behavioral monitoring. This information is also unique and exclusive to that specific individual, and it is also “biometric,” although not necessarily aimed at personal identification but at internal or mental identity.

For this reason, I propose that the criminal protection of human mental privacy should be grounded in three principles: (1) Its assimilation to other human organs; (2) the prohibition of unauthorized access, dissemination, or processing of the physical and/or psychological information, extracted during the immersive virtual experience, and (3) the prohibition of lucrative trafficking of this sensible personal information. In the line with the proposal of Bublitz and Merkel (2014, pp. 73–75), the brain hacking should be explicitly criminalized. Moreover, the mass trafficking of individuals’ mental data, whether identified or identifiable, must be prohibited, even in cases where the affected individuals have given their consent. Informed consent may exclude the individual protection under criminal law, but it does not dismiss the need to protect society, as it is done with drugs and other human organs trafficking and with child pornography, e.g.,

Regarding the question of who should be held criminally responsible, I argue it should primarily rest with the platforms responsible for collecting and storing the data withing their structure and cloud. They should be obligated to manage the risks they contribute to generating, arising from the content and services they provide and derive benefit from, whether directly or indirectly. It is essential to establish a system of criminal responsibility for these entities, in addition to potential liability for any other actor, whether a natural or legal person, who intentionally violates the privacy of other users, such as through device hacking and theft of the user’s mental data.

### 3.2. Risk 2: “Emotional harm” from immersive experiences

The second specific risk of virtual reality is its capacity to reproduce the “physical” experience of the avatar in the brain of the user. The brain perceives and processes the immersive experience “as if it were real,” making the user “feel it as real,” even though they know it is not (Slater et al., 2006; Slater, 2009). This characteristic allows the user to feel the experience of being a victim of a crime, retain memories of it, and may even transfer their traumatic experience to real life. When victims, for example, of sexual abuse have described their experiences, they refer to an “emotional harm” that “feels very real” (Belamire, 2016).

These types of criminal behaviors are foreseeable and will be likely frequent in virtual worlds, as these are multi-user immersive environments where individuals can have life-like interactions with each other and with virtual objects through their avatars in real-time. Such environments are a recreation of offline social life and reproduce both the good and the bad of it, especially in user-generated contents or socializing spaces, where actions are solely driven by users. The user can experience physical abuse, harassment, or become a victim of financial fraud, for example. However, it must be emphasized again that personal harm will only occur in fully immersive experiences (Castro, 2022). Therefore, the legal assessment of behaviors during virtual reality experiences will depend on the level of immersion of the user-victim, and the awareness that the other user has of it, which adds a unique complexity to the different situations that may arise. Similarly, regarding responsibility, it will be crucial to determine who created and/or controls the harmful content or who engages in the harmful conduct (user, platform owner), depending on the nature of the unlawful act.

#### 3.2.1. Physical and psychological harm from virtual experience

When considering the issue of physical (or psychological) harm to specific users that can result from immersive virtual experiences, three different situations can arise: (1) Individual harm resulting from the use and abuse of this technology by consumers according to its intended purpose; (2) Physical harm (injury) caused to the user by the deliberate actions of a bad actor; and (3) Physical or psychological harm to the user through actions performed on their avatar. The latter are specifically related to immersive VR.

The fundamental principle from a criminal policy perspective, in my opinion, is that the legal assessment of bodily harm, caused in the virtual space, should be approached in the same way as in the real world, considering the actual harm inflicted in the physical realm. What is illegal in real life, must be also illegal in the immersive virtual context. Attention should be given to the real harms and risks that can occur to the user (not necessarily what is reflected in the avatar) and they should be addressed in accordance with the general rules concerning crimes against life and the health of individuals.

(1) Physical (and psychological) harm to the user resulting from the use and abuse of virtual reality. As we have concluded in section 2, the long-term effects of prolonged and recurrent immersion in VR are still unknown and there is not enough consensus about them.

Similar to games and 2D technologies, the literature has reported potential physical or psychological harm that may arise in some individuals due to the abusive use of VR, as a product made available to consumers within its intended purpose. Reported harms include: cybersickness (Tian et al., 2022a), addiction (Paquin et al., 2023), dissociation from reality and depersonalization (Peckmann et al., 2022), body dissatisfaction or dysmorphia (Park and Kim, 2022), personal injuries due to accidents, and potential specific or heightened risks for children and adolescents (Miehlbradt et al., 2021).

Nevertheless, as anticipated, the literature also shows that the results are contradictory and more research is needed (Kaimara et al., 2021). Similarly, Madary and Metzinger (2016) conclude that additional ethical deliberation will be also required to mitigate risks and increase awareness among the general public about VR users. The review conducted by Paquin et al. (2023) integrates existing evidence on mental health in users of video games, social networks, and the metaverse, aiming to anticipate foreseeable risks. The main conclusion is the challenge of drawing general conclusions, as the adaptive use of these new technologies can also bring clear mental health benefits. The impact of excessive use is heterogeneous and influenced by factors such as technological motivation, level of personal development, sociodemographic context, and previous history of mental health problems.

However, the accumulated evidence does allow for the anticipation of risks that have already been documented in relation to video games and mass media, which could potentially be amplified with immersive virtual reality or the metaverse. As stated by Spiegel (2018), while the extent of risks to mental health from VR is not yet clear, considering the known issues with 2D gaming, it is expected that they are at least equally significant. In the same vein, The U.S. Surgeon General’s Advisory (2023) suggest similarly cautions.

Considering the aforementioned, in my opinion, these cases should currently be addressed in accordance with general principles of product responsibility and caution. Industry should be required to implement effective risk control measures, with enhanced protection for children (Miehlbradt et al., 2021) and other vulnerable groups. However, this control of uncertain risks for public health should be carried out, at present, outside the realm of Criminal Law, which should only intervene when there is a clear consensus regarding the potential long-term harm of immersive VR. I also believe that research in this field, shared with persuasive AI, should be prioritized, given the systemic nature of the potential risks implied for public health.

(2) Physical harm to the user caused directly by the deliberate actions of a third party (bad actor). This can occur, for example, through the hacking of their devices, which is expected to be more feasible in multi-user VR social environments characterized by high connectivity, numerous technical vulnerabilities, and a rush to join and participate in the metaverse (Vondráček et al. 2023, p. 2). On example is the so-called Human Joystick Attack (Casey et al., 2021) or the malicious use of the Virtual-Physical Perceptual Manipulation (VPPM) system, which can cause accidents or physical harm to the user through unconscious manipulation of their spatial perception. For instance, redirected walking can unconsciously guide a user toward a staircase where they may fall. In the case of swapping, the head-mounted display recognizes people around the user and incorporates them into the game as enemies, potentially resulting in the user physically attacking them (Tseng et al., 2022). This category of physical harm also includes cases of emotion hacking, by accelerating the user’s heart rate with a terrifying experience. If any physically harmful outcome occurs, it could be subject to punishment under general rules, with the virtual environment being irrelevant.

(3) Physical harm inflicted on the avatar, with repercussions on a specific user of the immersive virtual experience. This specific group of cases in VR, associated with its immersive nature, involves conduct performed within the virtual environment and directed toward the avatar, which represents the user. For example, consider a scenario where one avatar punches another avatar in the face or stabs it with a knife. The question arises as to whether these behaviors, while not causing actual physical harm to the user but potentially causing a significant emotional distress, could be equated. The obvious answer seems to be no, even in the context of fully immersive experiences with full-body haptic devices. In case of homicide or injury offenses, the punishment is imposed for the actual death or physical harm to a human being and this, simply, does not occur when, for example, someone is made to experience their own death through hacking (Qin et al., 2022) or consent to it, as proposed by Oculus founder Luckey (2022). Likewise, no physical injury occurs to the user’s body when they are made to experience the pain of a stabbing, for example, through a full-body haptic suit.

However, one might question whether such behaviors could give rise to psychological harm and be legally punished as psychological injury. In my opinion, while theoretically possible, it does not appear feasible in practice, at least not currently. To establish the existence of psychological injury in a legal proceeding, substantial evidence of non-preexisting psychological harm and identifiable biomarkers (clear and specific neuro-psychological expression) would be required, which traditionally presents significant evidentiary obstacles. Moreover, it would be necessary to establish a causal link between the psychological injury and the specific virtual interaction experienced. In other words, the psychological harm must directly result from interacting with potentially harmful virtual content or deliberate actions of a third party through their avatar. Additionally, further research and consensus are required to determine the exact nature of psychological harm that may arise from immersive VR, including the identification of biomarkers, the potential severity of such harm, and individual variations that may influence its effects. These factors present challenges in substantiating psychological harm in a criminal trial, considering the rules of evidence and the standards for scientific proof.

#### 3.2.2. Violation to personal freedom or autonomy: sexual assaults

Can that reported “emotional harm” serve as basis for the specific harm inherent in offenses against personal freedom and autonomy? The answer is clearly yes. In fact, one of the personal offenses that has gained significant social relevance, due to its prevalence, is online sexual harassment, which is not surprising, given the amplifying characteristics of the 2D and 3D digital environment compared to the physical realm. According to a 2021 report by the Pew Research Center (Vogels, 2021), it was noted that four-in-ten Americans have personally experienced online harassment, particularly among adults under 30. Furthermore, in the context of sexual harassment, which is prevalent among female users, it has doubled since 2017. Thirty-three percent of women under 35 report having been sexually harassed online, compared to 11% of men under 35. Some other surveys indicate that 49% of female users and 36% of male users have encountered some form of sexual harassment (Carson, 2018).

In relation to criminal offences against sexual freedom in virtual spaces, the first notable case was the LambdaMOO case that occurred in early 1993 in a rudimentary text-based virtual world. Dibbell (2005) described the incident as “phantom sexual violence,” involved a user named Mr. Bungle who created a subprogram allowing him to control other players’ characters without their consent, forcing them to engage in non-consensual sexual acts with each other through a “voodoo doll” mechanism. In 2016, Jordan Belamire reported an incident of sexual abuse in the multiplayer mode of the game called QuiVr, where players assume the role of an archer shooting down the walking dead. Shortly after starting to play, despite the only distinguishing factor between players being their voices, the player “BigBro442’s disembodied helmet faced me dead-on. His floating hand approached my body, and he started to virtually rub my chest... This goaded him on, and even when I turned away from him, he chased me around, making grabbing and pinching motions near my chest. Emboldened, he even shoved his hand toward my virtual crotch and began rubbing. There I was, being virtually groped in a snowy fortress with my brother-in-law and husband watching..”. “Remember that little digression I told you about how the hundred-foot drop looked so convincing? Yeah. Guess what. The virtual groping feels just as real. Of course, you are not physically being touched, just like you are not actually one hundred feet off the ground, but it’s still scary as hell” (Belamire, 2016). This group of cases could also include hypothetical scenarios involving non-consensual sexual contact through teledildonics, which are devices used for remote sexual activity, including remote mutual masturbation. As noted by Dremliuga et al. (2019), an unauthorized intruder could potentially hack such devices and engage in intimate contact without consent, highlighting the technical insecurity often present in such devices and hardware.

These behaviors, if performed in the physical realm, would be classified as sexual assault or abuse, depending on how each legal system treats non-consensual sexual contact without violence or intimidation. Regardless of their specific legal classification, my argument is that these criminal behaviors should be treated similarly to real-world offenses, even if they occur in a virtual or metaverse space. In legal systems such as Spanish law, sexual assaults are considered offences against sexual freedom, characterized by the absence of consent and the commission of a sexual act involving the victim. Subsequently, under Spanish Penal Code (SPC), the specific offense and its corresponding punishment are determined based on factors such as the means employed (such as violence, intimidation, or abuse of authority) and the severity of the behavior (e.g., rape) (Art. 178 and 179 SPC). Typically, in the case of adult victims, direct physical contact between the victim and the perpetrator is also required (not required for victims under 16, as provided in art. 181 SPC). In the absence of direct physical contact, the act may be punishable either as a general offense against freedom (harassment) or as degrading treatment.

In the case of virtual reality, apart from the evidentiary problems, the central issue is to determine whether the interaction that occurs between avatars (virtual contact) can be considered “physical” contact between the users behind them (also the very illustrative work of Lemley and Volokh, 2017, 127 ff.). In my opinion, this question should be answered affirmatively. The contact that takes place between VR users can be regarded as physical in nature, because it is sensorially perceived through the headset, speakers, and other haptic devices such as vests and gloves. Additionally, the brain itself attributes a physical nature to the experience through the immersive environment and emotional evocation. In immersive VR, the bodily impact transforms into a cerebral representation, a mental experience of the real world through recreation. In the specific case, as has been pointed out, the legal treatment is contingent upon the level of immersion experienced by the victim and the perpetrator’s awareness thereof. For instance, touching the victim’s chest in a 2D virtual experience may not be considered sexual assault in a strict sense, and only potentially sexual harassment should be discerned. However, in a 3D immersive experience, the same behavior could be deemed sexual assault, the severity of which would depend on the specific circumstances surrounding the act and the criminal intent (*guilty mind*) of the perpetrator. Ultimately, attention must be given to the type of harm and the severity it has caused to the user in the physical real world.

Regarding online or VR sexual harassment or abuse, it is common for their significance or severity to be underestimated, especially because there is no “physical touch” involved, and it is argued that simply disconnecting would have been sufficient for self-defense, avoiding the continuation of the non-consensual experience. For instance, in 2021, immediately after Meta announced the launch of its social virtual reality platform (Horizon Worlds), a beta-testing group user reported being groped by a stranger (Basu, 2021). In an internal report, Meta stated that the tester should have activated the “Safe Zone” tool, which allowed the creation of a personal bubble, preventing another user from speaking to you, touching you, or interacting with you in any way (Heath, 2021).

Mary Anne Franks has aptly highlighted to the frequent contradiction in discourse surrounding these types of technologies. On one hand, when discussing their potential positive impact, such as in mental health therapy or education, the discourse focuses on their “reality” and efficacy. However, when discussing the negative effects they can have, they are dismissed as “unreal,” and the voices of the victims, particularly in cases of sexual nature, are discredited (Franks, 2017). Similarly, Wiederhold (2022, pp. 479–480) has emphasized that 3D technology is more dangerous precisely because of its immersiveness and because the trauma related to the virtual reality experience can carry over to the real world. For example, those who experience virtual sexual assault will most likely experience an increase in heart rate and other physical measures of anxiety—the same fight or flight response that they would have if the incident had happened in the real world. As a result, negative virtual experiences can impact people psychologically, physically, and socially, even when offline. It is not easy simply to take the headset off and forget the experience.

Indeed, in multi-user virtual spaces, it is necessary for platforms to provide users with self-protection measures and effective means of identifying users behind avatars (digital ID). These measures should be established by default. Moreover, this need becomes directly indispensable, considering that the main challenge in prosecuting crimes committed in virtual environments lies in their effective enforcement. As Lemley and Volokh (2017, p. 128) asserts: “The ability to define default consent in software can make VR safer than the real world—for instance, well-designed software may let me consent in advance to certain types of touching but not others, or touching by some people but not others, and touching that is not consented to will not even be felt. The question that remains is to what extent, if at all, we should view it as the victim’s job to set software consent boundaries; more on that below.”

Victims must take precautionary measures for their own interest in any context. However, the law should not focus on the victim’s behavior, but rather on the non-consensual actions of the aggressor. Just like in the real world, the criminal protection of victims does not depend on their effectiveness in self-defense. This understanding should not disregard the modulation or exclusion of liability for the offender in cases where the victim voluntarily puts themselves in danger, assuming a highly probable risk of harm. Victims are not expected to, for example, carry and use self-defense tools when running in a relatively isolated area to be legally protected in the event of a robbery or assault. Therefore, it is not reasonable to consider the committed criminal behavior in the context of virtual reality as irrelevant simply because the victim did not activate a personal distance bubble or disconnect. Naturally, most behaviors will be somewhat surprising and of short duration because the victim can disconnect from the experience. However, even if they are sudden and brief attacks, they can still constitute sexual abuse or, at the very least, sexual harassment. Any other approach would shift the focus onto the victim rather than the aggressor, which is unacceptable.

The law must govern in all spaces of social interaction, and virtual spaces or the future metaverse cannot be exceptions. What is unlawful in the physical world must also be unlawful in the 2D and 3D digital environments, subject to punishment under criminal law in accordance with general rules. In cases of sexual offenses, the core of the offense involves engaging the victim in direct, non-consensual sexual contact, which occurs within the immersive experience through avatars. The required interaction between perpetrator and victim, as well as the nature of the behavior committed, also occur in the virtual environment. This intermediate “spatial” scenario, unlike 2D, allows for necessary direct contact between the perpetrator and the victim, even if it is not strictly physical contact as in the real world. Moreover, other illicit behaviors in the 2D environment, such as verbal harassment, defamatory content, hate speech, or the depiction of child pornography through avatars, can also be subject to legal sanctions. The challenges lie in matters of jurisdiction, identifying responsible parties, and enforcing the law, rather than in the definition of criminality, indeed.

In conclusion, the implementation of industry defense measures by design and the availability of self-defense security measures for victims should not exempt the punishment of criminal acts committed in virtual environments. The challenges in prosecuting these crimes or others should not be used as justification to invalidate the applicability of criminal law in such spaces.

### 3.3. Risk 3: Manipulation of human behavior

Immersive virtual reality, as an emotion-inducing neurotechnology, also carries a clear risk of citizen manipulation. We are referring here to a direct risk of manipulation, through the immersive experience and temporally connected to it, with the aim of inducing ideas or directing user behaviors. It is not, therefore, an analysis of the long-term risk of enduringly transforming the user’s mental integrity or generating such delayed changes through the accumulation of virtual reality use. As mentioned, such risks are currently unknown. In essence, we focus on the creation of risks aimed at instigating ideas or behaviors in the user, utilizing non-consensual manipulation, which would be closely related to the concept of *‘inductive deception*.’

This risk would be greater than that of persuasive algorithms in the 2D environment of social media, which have already proven to be a serious danger to the mental health of our adolescents, consumers, and democracy (Harari, 2018, 2019). As highlighted by Heller and Bar-Zeev (2021, p. 10), we are beginning to face the social harm caused by persuasive algorithms in social media, influencing political discourse and affecting the mental health of citizens. Persuasive AI feeds our emotions with content and uses addictive mechanisms to keep us hooked. But... “What happens if these impulses are combined—and augmented—by an alternative reality that we treat as real?”

As acknowledged in Title II of the AI Act (*Proposal for a Regulation of the European Parliament and of The Council laying down harmonized rules on artificial intelligence (Artificial Intelligence Act) and amending certain Union legislative acts 2021*), certain AI systems can generate unacceptable risks of manipulating individuals. For this reason, Article 5 prohibits the use of artificial intelligence systems that employ subliminal techniques or exploit the vulnerabilities of specific population groups, such as minors and people with disabilities, to materially distort a person’s behavior. However, this prohibition is limited to AI systems that could cause physical or psychological harm to a person, such as inducing suicide or self-harm. In Spain, for example, the public dissemination, through any means, of *content specifically aimed* at promoting or inciting such behaviors among minors and particularly vulnerable individuals due to disability has recently been criminalized as risk-based offences (Art. 143bis, 156ter and 189bis SPC). However, there is still ambiguity and a lack of a clear stance, regarding the treatment that persuasive AI should receive, especially when the algorithm is limited to suggesting content based on the user’s previous searches.

According to the provisions of the IA Act, it can be interpreted that the dissemination of such content in virtual spaces would be directly covered by this prohibition, as it involves technology mediated by underlying AI systems. Therefore, inducing suicide by a user in a virtual environment through their avatar should be treated according to general rules, just as it would be in the real world. For example, the case depicted in the Netflix series “Kiss Me First,” which takes place in a virtual space designed to induce user suicide, would be a prohibited practice that should be covered in the Criminal Code. Criminal liability should be attributed to the individuals or legal entities responsible for such content.

However, the AI Act excludes from this general prohibition other manipulative or abusive practices targeting adults and minors, if they have another purpose. It defers to existing sectoral legislation in the areas of data protection, consumer and user protection in digital services to cover such practices. With any other purpose, it is not either considered a “high-risk system” for citizens, because it is not included in the catalog provided in Title III. It merely states that legislation must ensure that individuals have adequate information, freedom of choice, and are not subjected to profiling or other practices that may affect their behavior.

Within the legislation to which the AI Act defers, although not explicitly mentioned, would also be the Criminal Law. Bublitz and Merkel (2014) have pointed out that, inexplicably, the non-consensual manipulation of individuals is not adequately covered by Criminal Law. Cognitive freedom (the right to freely shape one’s internal forum without unauthorized influence) is a concept that belongs to the current discussion on neurorights. However, an evident anchor can be found in the fundamental freedoms of thought and conscience (Bublitz, 2022; Ligthart et al., 2023). However, criminal codes generally do not include a generic offense that protects against the non-consensual manipulation of a person’s ideas. Protection has traditionally focused, in accordance with its liberal origins, on safeguarding ideological, conscious, or religious freedom against the powers of the State. Essentially, the freedom to exercise these rights publicly has been protected. This can be clearly appreciated in international declarations of human rights, which serve as the foundation and interpretive reference for national Penal Codes. It should be sufficient to mention, for example, Articles 18 and 19 of the Universal Declaration of Human Rights of 1948 (ONU-UDHR). The formation of thought, the activation of negative emotions, or the influence on decision-making processes are only fragmentarily protected by Criminal Law.

Using Spanish Criminal Law once again as an illustrative and generalizable example, the prohibition of indoctrination, whether consensual or not, is only applicable when it involves ideas and values considered socially unacceptable. This is known as “*hate speech*,” which glorifies or promotes terrorism, discrimination against individuals or groups, or pedophilia. On the other hand, affecting a person’s internal forum (inducing negative emotions or influencing their behavior) has traditionally been prohibited, if the perpetrator achieves their objective through illegitimate means. Such illicit means involve the victim’s lack of free consent or an attack on their dignity, such as coercion (violence, intimidation), abuse of superiority or vulnerability, harassment, degrading treatment, or deception. This can be observed in offenses such as threats, harassment, offenses against moral integrity, robbery, and fraud…

Therefore, outside of those cases, in Criminal Law, mere indoctrination or unauthorized manipulation would not be punishable. This criminal policy approach may need to be reevaluated in light of the current development of neurotechnologies and artificial intelligence, as advocated by Bublitz and Merkel (2014).

In the case of neurotechnologies in a narrow sense, which refers to devices with the potential to directly access and/or interfere with a person’s brain activity, I argue that any unauthorized direct interference, whether invasive or not, and regardless of its duration, should be explicitly criminalized in the Penal Code to protect the mental integrity of individuals. Without prejudice to its potential inclusion within the scope of generic offenses or as a crime against moral integrity, non-consensual alteration of brain activity (*brain hacking*) should be expressly defined as a criminal offense today, as technology has reached a level of development that enables such direct interference (González-Tapia and Isabel, 2022).

In the case of immersive virtual reality, the assessment becomes more complex because it is a non-invasive, indirect, and less effective technology.

(a) From the perspective of content moderation, concerning ideas or expressions, it is a intricate field, especially in the context of Criminal Law, where there is a simultaneous need to safeguard freedom of expression in a democracy. For example, verbal expressions or violent actions that may occur within the context of a game like Fortnite can be socially perceived in different ways in a context of free socialization or in a work environment, with some considering them as promoting violence or offensive expressions. If this poses significant challenges for content moderation on platforms, it also complicates the design of criminal policies related to such behaviors and the public dissemination of such content. Like real life, the context will largely determine the legal assessment of behaviors whose legal significance is not unambiguous and not clearly and manifestly unacceptable. For this reason, from a criminal policy perspective, penal protection should focus not so much on the content itself but rather on the target of the content or experience, safeguarding minors and vulnerable groups, as well as the means employed, in order to ensure the user’s informed and freely given consent. In my opinion, the current legal framework is sufficient in this regard.

(b) In terms of manipulative effectiveness, immersive VR represents an intermediate risk between neurotechnologies in the strict sense and persuasive means that can be developed in the real world (e.g., propaganda, marketing, indoctrination), in the 2D digital environment of social networks (affective AI), or in non-immersive VR situations. As mentioned, the specific risk presented by immersive VR stems from the synthesis of the psychological characteristics of virtual reality and the underlying persuasive AI systems. It lies in its ability to manipulate citizens through the emotional persuasion of affective AI, enhanced by the immersive nature of virtual reality. As an intermediate risk, it does not appear that the penal protection of users’ mental integrity should have the same extent as proposed for specific neurotechnologies. For this reason, I believe that, in addition to the recipients or means employed, Criminal Law should only consider specific sectors in which there is likely to be a greater impact or potential for more severe consequences.

Considering the accumulated experience regarding persuasive AI, the sectors where disinformation and manipulation of citizens have already demonstrated their harmful potential are democratic institutions and consumer protection, as well as the mental health of our adolescents (UNESCO, University of Milan-Bicocca, 2023, among others). Concerning immersive virtual reality, a similar impact can also be expected in these areas. Let us focus on the example of emotional manipulation for commercial purposes through immersive VR. In a recent risk report, the European Parliament (EPRS European Parliamentary Research Service, 2022) has highlighted the issue of direct marketing based on geolocation and emotional response. Users will be offered product selections based on their behaviors and reactions, which can be permanently, intensively, and in real time monitored throughout their VR experiences. In the metaverse environment, users may be increasingly subjected to subliminal, highly persuasive, and personalized advertisements: neurotargeting.

In my opinion, consumer protection against this risk should also be addressed through Criminal Law, as a reinforcement of the basic conditions established in sector-specific regulations. There is a clear consensus on the importance of psychological and neuroscientific knowledge in understanding the consumer decision-making process, in which emotions play a fundamental role. Thus, consumer decisions are not solely based on the characteristics of the product itself but also on the emotional connection that can be established with the product, the brand, and the purchasing experience. This is the basis of neuromarketing: applying neuroscientific knowledge to consumer psychology to influence their responses to the product through pleasurable emotions and arousal, particularly in advertising and customer loyalty (Russo et al., 2022). And for this reason, virtual reality and augmented reality are especially qualified environments for generating positive consumer attitudes toward the product, brand, and experience due to their immersive, interactive, and personalized nature (Kerrebroeck et al., 2017; Uhm et al., 2020; Dwivedi et al., 2023).

Heller and Bar-Zeev (2021) point out that VR-based advertising could be the epitome of advertising because its goal is to create ads that the recipient does not want to skip or may not even realize are ads, seamlessly integrated with experiences. If we add to this the fact that it will be personalized content (as consumer information is known) that perfectly aligns with their tastes, interests, and needs, it becomes advertising that is simply irresistible. Moreover, neuromarketing is based on the creation of consumer profiles that can even be applied to non-users of such technologies through predictive patterns enabled by artificial intelligence (P. Kellmeyer in UNESCO, University of Milan-Bicocca, 2023, pp. 39–40). Furthermore, the criminal assessment of this potentially exploitative personalized digital targeting must be considered, pondering a possible shift toward the metaverse in the near future, quantified by McKinsey and Company (2022) as a business that has already received over $120 billion in investments, with 79% of users making purchases, and projected to reach a business volume of $5 trillion by 2030.

On the other hand, it has also been said that immersive VR is an optimal instrument for *nudging* because decisions and emotions go hand in hand (Steinert and Friedrich, 2020). In this sense, it can be interpreted that immersive VR would fall under the definition of a “system for emotion recognition,” as stated in Article 3(34) of the AI Act, due to its ability to identify or infer emotions or intentions of natural persons based on their biometric data (eye-pupil tracking system). It is also a system capable of evoking emotions in the user in a subliminal manner.

Under Spanish Criminal Law, for example, individual consumers are protected through the offense of fraud, which addresses financial frauds involving deception (Art. 248 SPC). It is also provided, that consumers are protected collectively through the offense of false advertising (Art. 282 SPC). This legal framework may be adequate for addressing untrue features made by manufactures or traders, such as those related to commercial devices utilizing neurostimulation or virtual reality, when false claims are made about their effectiveness. However, this socio-economic offense should be expanded to include subliminal advertising as a prohibited commercial practice, specifically referring to advertising that operates below the consumer’s conscious threshold. If consumers’ financial interests are protected against deception regarding the characteristics of the products offered in the market, it is perfectly comparable to protect them against the potential harm of illegitimately induced purchases. Thus, in my opinion, in light of these affective technologies, consumers should have the right to be openly informed that immersive VR experiences serve a commercial purpose and should be protected against subliminal techniques that go beyond deception and circumvent the provision of informed and voluntary consent.

## 4. Conclusion

This article has examined the role that Criminal Law should play in regulating the non-therapeutic use of immersive VR, specifically its widespread use by consumers and users. We begin by considering immersive VR as an intermediate risk scenario, between criminal activities occurring solely in the physical world and those emerging in 2D digital environments (cybercrimes and criminal behavior associated with social networks and persuasive AI).

Firstly, we have analyzed specialized literature to determine the nature of virtual reality. Technically, virtual reality is a neurotechnology infused with high-risk artificial intelligence. Functionally, VR is a “transformative” neurotechnology capable of altering individuals’ perception of reality. Its effectiveness lies in emotionally immersing users in virtual experiences, akin to how our brains function. Consequently, the immersive nature of virtual reality gives rise to three specific areas of legally unaddressed risks: (1) the comprehensive nature of data collected and stored during its usage; (2) its ability to mentally replicate the physical experiences of avatars in users; and (3) its significant potential to manipulate individuals.

Secondly, the paper has briefly assessed both the reported cases and the foreseeable criminality in virtual worlds or “proto-metaverse,” focusing on the three risk areas and exemplifying them with attacks on mental privacy, sexual freedom, and consumer manipulation. Finally, it has been proposed that Criminal Law should promptly define the “red lines” of massive use of VR by citizens, with a democratic and human-centered approach. In line with this, a basic legal framework has been outlined for the criminalization of specific risks associated with immersive VR.

Regarding user privacy, we have proposed that the user’s mental information (beyond neural data) should be protected to the same extent as other human organs. This entails: (1) prohibiting unauthorized access, disclosure, or processing of the physical and/or psychological information extracted during immersive virtual experiences, and (2) prohibiting the profitable trafficking of this sensitive personal information. Regarding emotional harm arising from the virtual experience, it is essential to consider that its legal treatment directly depends on the level of immersion of the experience, resulting in a plurality of scenarios with variable significance. Therefore, the guiding criterion that can be extracted is to consider the real-life identity of the user-victim and user-perpetrator and the actual harm suffered, regardless of the avatars ‘representation in the virtual experience. In cases involving crimes against sexual freedom, their legal treatment may range from sexual abuse in highly immersive experiences (known by the perpetrator), to cases of mere harassment or mild degrading treatment. Finally, about the risk of human manipulation, it has also been concluded that it is difficult to institute generic offences, which should be provided for in relation to neurotechnologies in the strict sense instead. In relation to immersive VR, criminal protection must be partial and focus on specific sectors in which there is likely to be a greater impact or potential for more severe consequences. For example, with respect to consumer manipulation, the explicit prohibition of subliminal advertising, which can easily be carried out through immersive VR, has been proposed.

The criminality associated with immersive VR presents a complex landscape for Criminal Law. Factors such as the dual use of this technology, varying levels of immersion, defendant’s *mens rea*, IA-avatars, identifying authorship behind the avatar, lack of societal awareness regarding VR associated risk, jurisdictional and law enforcement challenges, and the liability of entities involved in virtual reality infrastructure, content ownership, or deriving benefits from virtual reality enterprises contributes to the complexity. In essence: a “gray zone,” at the crossroads of the liberal boundaries of legitimate intervention by Criminal Law, with systemic and diffuse risks that affect fifth-generation rights (neurorights).

## Data availability statement

The original contributions presented in the study are included in the article/supplementary material, further inquiries can be directed to the corresponding author.

## Author contributions

MIG-T: Writing – original draft, Writing – review & editing.
